# Multiscale tribology analysis of MHD hybrid nanofluid flow over a curved stretching surface

**DOI:** 10.1039/d3na00688c

**Published:** 2023-12-01

**Authors:** Khursheed Muhammad, Bilal Ahmed, Mohamed Sharaf, Mohammad Afikuzzaman, Emad A. Az-Zo'bi

**Affiliations:** a Department of Humanities and Sciences, School of Electrical Engineering and Computer Science (SEECS), National University of Sciences and Technology (NUST) Islamabad Pakistan khursheed.muhammad@seecs.edu.pk; b School of Energy and Power Engineering, Jiangsu University Zhenjiang China; c Industrial Engineering Department, College of Engineering, King Saud University P. O. Box 800 Riyadh 11421 Saudi Arabia; d UniSA STEM, University of South Australia Adelaide SA 5000 Australia; e Department of Mathematics, Mutah University Al Karak Jordan

## Abstract

In this study, we investigate the interactions of a hybrid nanofluid on a curved surface that is being stretched. The magnetic field is perpendicular to the flow and interacts with a mixture of molybdenum disulfide and argentum nanoparticles suspended in pure water, forming a hybrid nanomaterial. Our investigation considers heat transport analysis under different conditions, such as magnetohydrodynamic, Darcy–Forchheimer porous medium flow, Joule heating, and a convective boundary condition. We employ numerical and statistical methods to study the problem's intricacies comprehensively. Our findings indicate that Darcy–Forchheimer flow includes viscous and inertial forces, which results in higher flow rates and reduced skin friction. Additionally, the convective boundary condition leads to uniform temperature distribution within the hybrid material due to rapid internal heat transfer relative to surface resistance, significantly increasing the heat transfer rate.

## Abstract 2

In this study, we investigate the interactions of a hybrid nanofluid on a curved surface that is being stretched.

## Graphical abstract

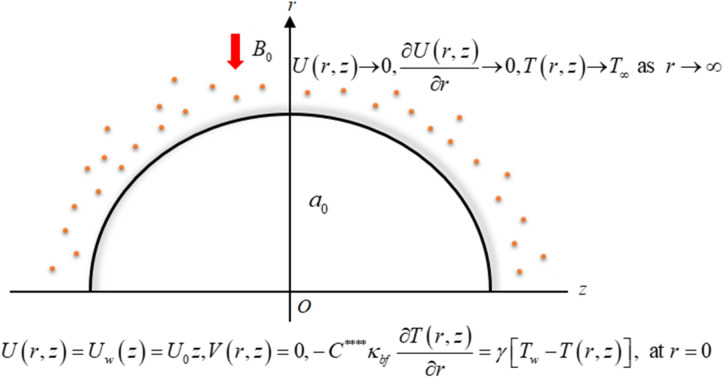

## 1. Introduction

Hybrid nanofluids are advanced colloidal suspensions that combine nanoparticles from different materials to create unique and synergistic properties. Incorporating different nanoparticles involves blending these nanoparticles to create a specialized nanofluid with enhanced thermal, electrical, and other functional characteristics. Lee *et al.*^1^ reviewed conflicting findings on the thermal conductivity of nanofluids. The study highlighted experimental milestones, proposed mechanisms and models, addressed data inconsistencies, and suggested directions for future research. The aim was to optimize nanofluids for enhanced thermal properties in various applications. Lee *et al.*^2^ developed surfactant-free zinc oxide nanofluids using a pulsed wire evaporation method in ethylene glycol. Five labs conducted round-robin tests to measure the thermal conductivity of three samples using in-house setups and a commercial device. Choi *et al.*^3^ discussed nanofluids' internal forced convective heat transfer attributes with experimental features. Nadeem *et al.*^4^ inspected hybrid nanomaterial distribution by considering two distinct nanoparticles in pure water over a curved surface. Waqas *et al.*^5^ numerically computed melting heat transfer in the nonlinear radiative flow of hybrid nanofluids due to permeable stretching curved surface. Wahid *et al.*^6^ explored the flow and heat transfer of a hybrid nanofluid induced by an exponentially stretching/shrinking curved surface. More interesting studies regarding nanofluids are addressed in ref. 7–22.

Regression analysis is a statistical technique used to establish empirical correlations between parameters. It has practical applications in fluid mechanics, allowing researchers to understand, predict, and optimize various fluid behaviors. These correlations are crucial for characterizing flow patterns, predicting pressure drops, and modeling turbulence in complex fluid systems. It also supports the analysis of experimental data and validation of numerical simulations and helps to design efficient systems while making informed decisions in fluid mechanics. Moreover, quadratic and multiple quadratic regression analyses are specialized approaches that can accommodate linear and non-linear relationships between variables and establish predictive models that enhance the understanding and manipulation of fluid behavior. Najm^23^ examined polynomial chaos methods for probabilistic uncertainty quantification in computational fluid dynamics (CFD) predictions. The study reviewed various CFD applications and challenges, such as flow in porous media, incompressible and compressible flows, thermofluid and reacting flows, and cross-cutting challenges related to time unsteadiness and longtime horizons. Zhang *et al.*^24^ presented an improved airfoil design using a modified shape function transformation. The design was validated through computational fluid dynamics and experimental testing. Optimization was performed using a modified multi-island genetic algorithm combined with non-linear programming. The resulting optimized airfoil demonstrated enhanced performance and lift-to-drag ratio, providing insights for future airfoil design. Kumar *et al.*^25^ employed spectral relaxation to investigate nanofluid flow over a porous stretching sheet, considering slip, mixed convection, dissipations, and nanoparticle control. The study discussed velocity, temperature, and concentration graphs; verified prior findings; conducted regression analysis for local Nusselt numbers; and highlighted the impact of thermophoretic and Eckert number factors on diffusion. Liu *et al.*^26^ used quadratic regression orthogonal combination (QROC) and a genetic algorithm (GA) to optimize the coal pyrolysis filtration system's performance and extend the filter tube lifespan with compelling predictions and low mean square error. References are provided for studies discussing issues associated with regression analysis (ref. 27–33).

The significance of this study lies in examining the combined effects of molybdenum disulfide and argentum nanoparticles suspended in a water solution over a curved surface, influenced by an inward magnetic field, magnetohydrodynamics, Darcy–Forchheimer porous medium flow, Joule heating, and a convective boundary condition. This yields insights into intricate heat transport interactions through a hybrid nanomaterial and porous medium. The reason we have combined molybdenum disulfide (MoS_2_) and silver (Ag) nanoparticles is their versatility and compatibility with different nanomaterials (their mixture can be customized to achieve specific results). The investigation employs numerical and statistical methodologies for a comprehensive study of the intricacies of the problem. The findings of this study hold relevance in various applications such as advanced thermal management systems, nanoparticle-enhanced heat exchangers, and innovative cooling technologies where the combined effects of molybdenum disulfide and argentum nanoparticles suspended in a water solution can be harnessed to optimize heat transfer efficiency and system performance.

The article was structured as follows: Section 2 encompassed mathematical modeling, Section 3 presented regression analysis, Section 3 contained a discussion of results, and Section 4 offered a summary of the findings.

## 2. Modeling of the problem

**Fig. 1:**
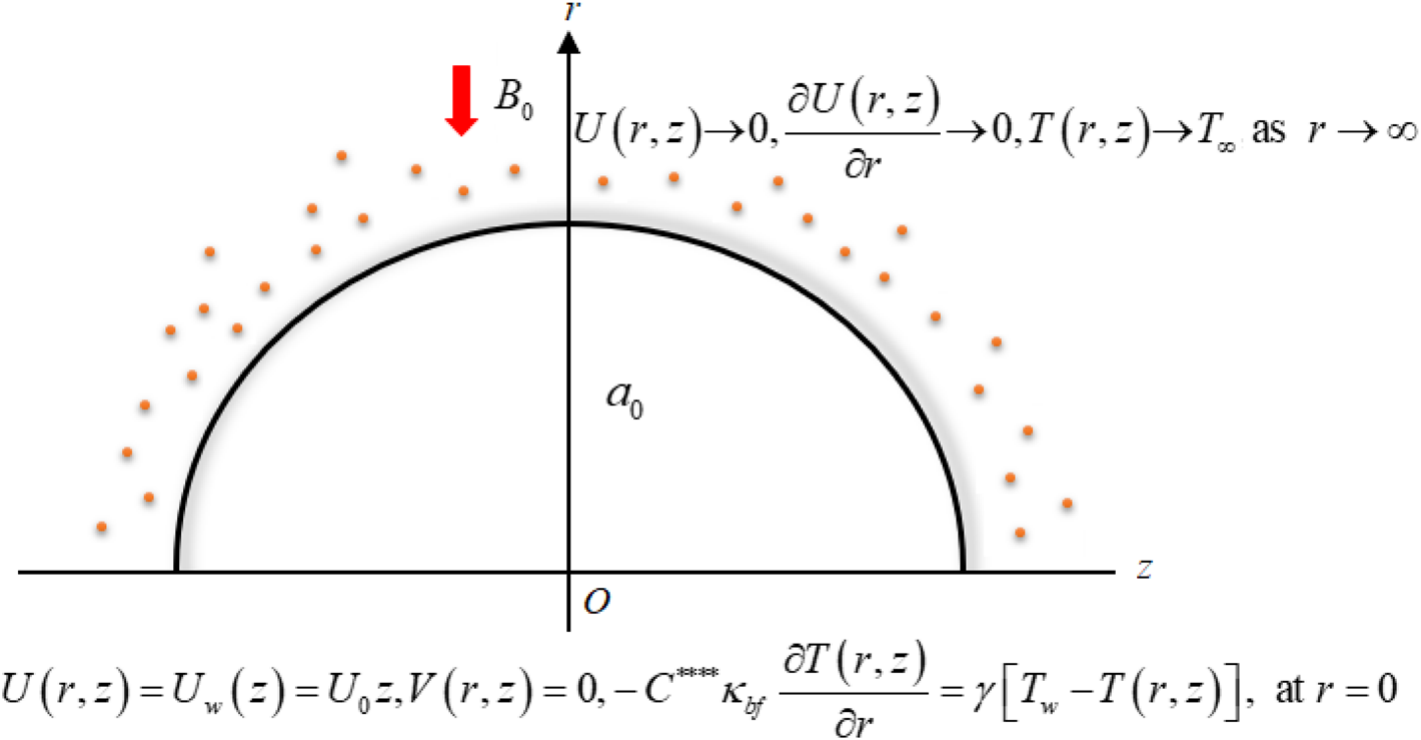
Geometrical description.

A two-dimensional flow of a hybrid nanofluid is considered over a curved surface with a radius “*a*_0_,” which is undergoing stretching. The problem is formulated in curved coordinates (*r*,*z*), and an applied magnetic field of intensity “*B*_0_” with an inward perpendicular direction to the flow is present. This field interacts with a mixture of molybdenum disulfide (MoS_2_) and argentum (Ag) nanoparticles suspended in a pure water (H_2_O) solution, creating the hybrid nanomaterial. The velocity field is *V* = [*U*(*r*,*z*), *V*(*r*,*z*), 0]. The analysis encompasses heat transfer effects while accounting for magnetohydrodynamics, Darcy–Forchheimer porous medium flow, Joule heating, and a convective boundary condition. The investigation involves both numerical and statistical approaches to study the problem comprehensively. Fig. 1 provides a visual representation of the problem's geometry, while Table 1 offers essential thermophysical values for both the nanoparticles and the base fluid.

**Table 1:** Nanoparticle (MoS_2_ and Ag) and base fluid (H_2_O) thermal features

Materials/characteristics	*ρ* (kg m^−3^)	*κ* (W m^−1^ K^−1^)	*σ* (S m^−1^)	*c*_p_ (J kg^−1^ K^−1^)	Pr
H_2_O	997	0.613	5.5 × 10^−6^	4179	6.2
Ag	10 490	429	6.3 × 10^7^	235	—
MoS_2_	5060	34.5	2.09 × 10^4^	397.746	—

By incorporating the assumptions mentioned above and considering conditions such as negligible viscous dissipation and the application of boundary layer approximations (*O*(*U*) = *O*(*z*) = *O*(*ρ*_bf_) = *O*(*ρ*_hnf_) = *O*(1), *O*(*V*) = *O*(*r*) = *O*(*δ*), and *O*(*μ*_hnf_) = *O*(*μ*_f_) = *O*(*δ*^2^)), the following set of PDEs can be derived (following ref. 7):1
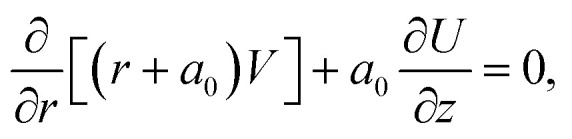
2
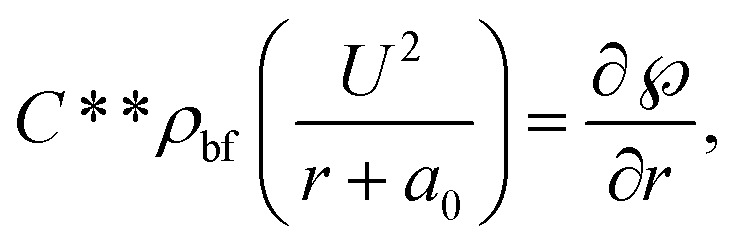
3
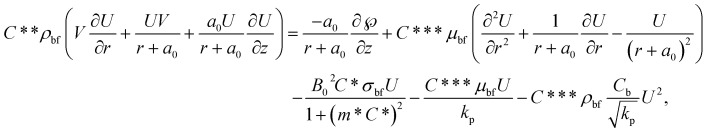
4
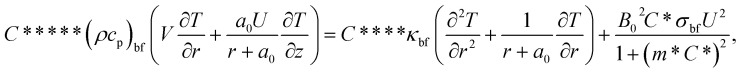
with5
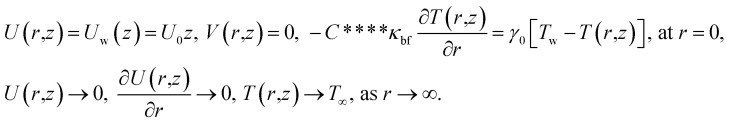


For hybrid nanofluid the electrical conductivity is defined by6
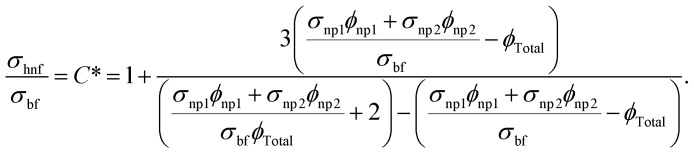


Hybrid nanomaterial density by the Xuan and Li model is7
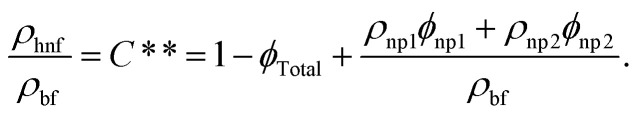


Dynamic viscosity *via* the Brinkman model for the hybrid nanomaterial is8
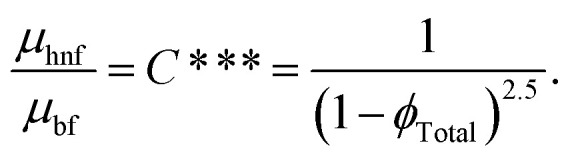


The Maxwell model defined thermal conductivity of the hybrid nanomaterial using9
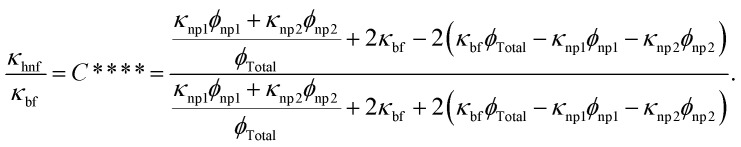
10
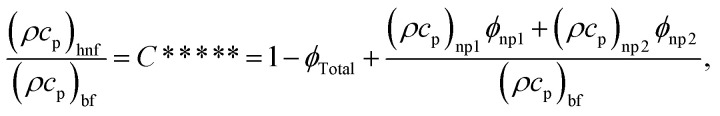

11 *ϕ*_Total_ = *ϕ*_np1_ + *ϕ*_np2_.

For the conversion of flow as mentioned above and heat transfer related PDEs and associated BCs into non-dimensional form, we introduce the following dimensionless non-similar variables (following ref. 7).12
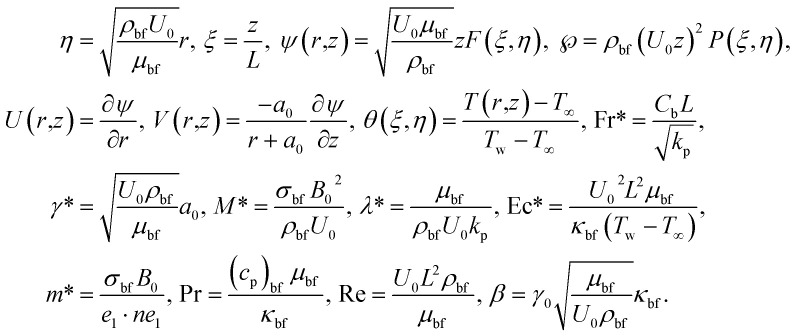


After making use of the associated velocity component in terms of stream function, we get eqn (1) identically satisfied, and the rest of the equations (eqn (2)–(5)) take the following form after using first-order truncation 
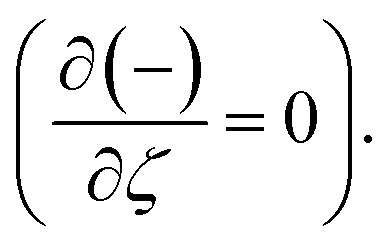
:13
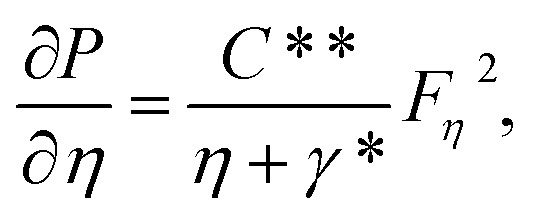
14
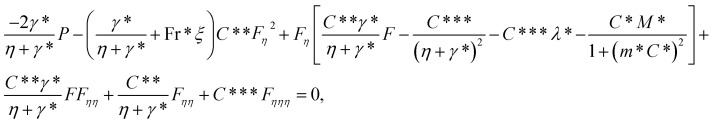
15
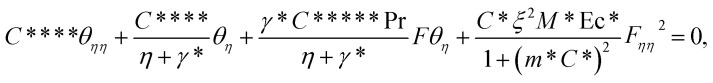


Eliminating *P* from eqn (13) and (14), we get16
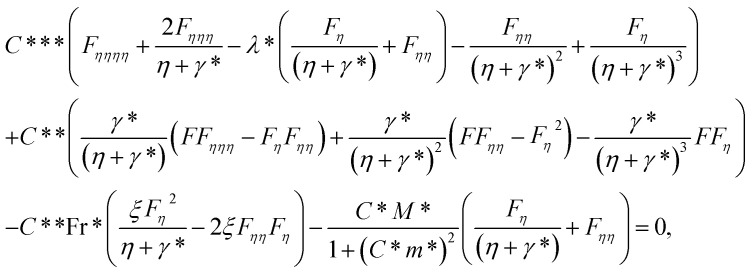
17
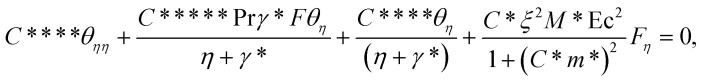
with BCs18
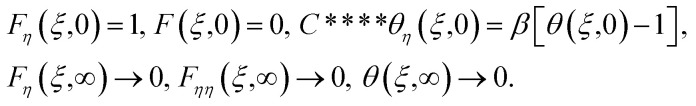


Dimensional skin friction 
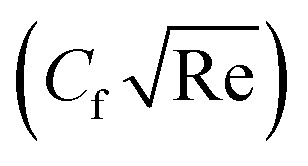
 and Nusselt number 
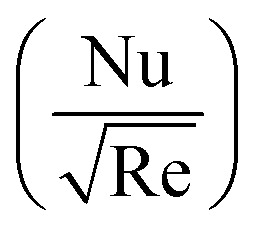
 are (following ref. 7)19
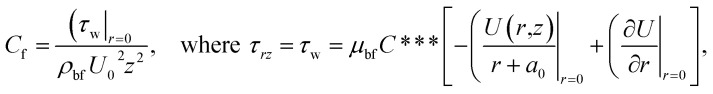
20
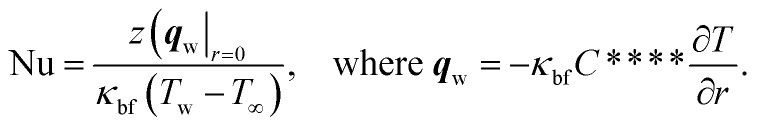


After using appropriate substitutions in eqn (19) and (20), we get non-dimensional 
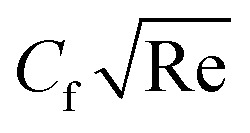
 and 
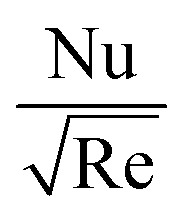
 as21
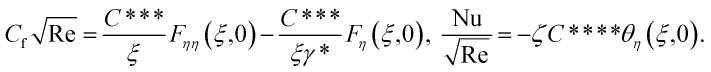
Here 
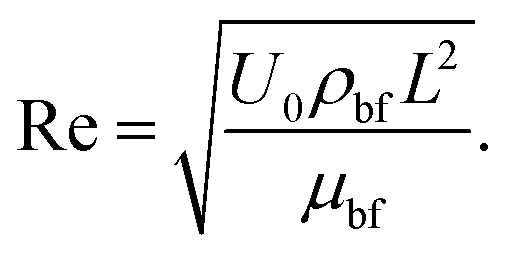


## 3. Regression analysis

To study the comparative effectiveness of two physical parameters on the skin friction 
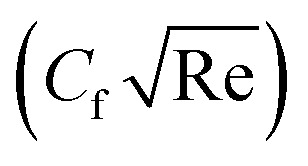
 and Nusselt number 
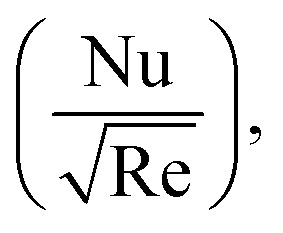
 we have used the multiple quadratic regression model. A multiple quadratic regression model is a statistical technique for modeling the relationship between a dependent variable and one or more independent variables. For this purpose we have taken 51 values of λ* and Fr*, such that λ* ∈ [0.1,0.5] and Fr* ∈ [0.1,0.7]. In the case of regression analysis of 
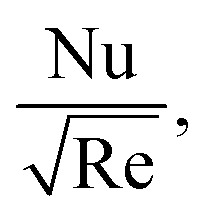
 we have chosen 51 values of Ec* and *β* such that *β* ∈ [0.1,0.5] and Ec* ∈ [0.1,0.3]. During these analyses all other physical parameters are kept fixed. The multiple quadratic regression model for skin friction 
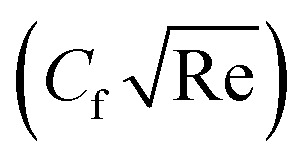
 and Nusselt number 
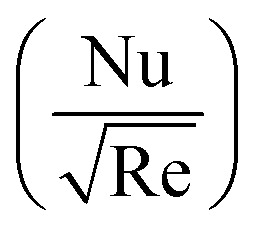
 is defined according to Kumbhakar *et al.*^33^

22 (*C*_f_)_Predicted_ = *C* + *c*_1_Fr* + *c*_2_λ* + *c*_3_(Fr*)^2^ + *c*_4_(λ*)^2^ + *c*_5_Fr*λ*,

23 (Nu)_Predicted_ = *N* + *d*_1_*β* + *d*_2_Ec* + *d*_3_(*β*)^2^ + *d*_4_(Ec*)^2^ + *d*_5_Ec*β.

In the above expression, (*C*_f_)_Predicted_ and (Nu)_Predicted_ represent the predicted skin friction and Nusselt number, *C*and *N* are intercepts, while *c*_1_, *c*_2_, *c*_3_, *c*_4_, and *c*_5_ and *d*_1_, *d*_2_, *d*_3_, *d*_4_, and *d*_5_ are regression coefficients.

## 4. Discussion

The primary objective of this section is to analyze the responses of flow (*F*_*η*_(*ξ*,*η*)), temperature (*θ*(*ξ*,*η*)), skin friction 
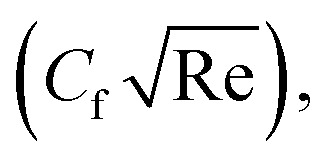
 and Nusselt number 
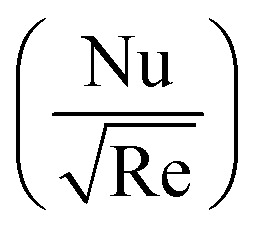
 with the variation of different physical parameters.

### 4.1 Discussion of methodology

The governing non-dimensional PDEs and their BCs are solved using the NDSolve numerical tool in Mathematica. It simplifies the solving process by automatically selecting the most appropriate method and controlling the adaptive step size, making it easier for users to solve complex differential equations with high accuracy. Similarity transformations are applied to simplify the PDEs, treating them as ODEs. NDSolve automatically selects a numerical method suitable for the equations and desired precision. The size of time or space steps is adjusted using NDSolve as the solution progresses, focusing computational effort where it is needed. Starting from the initial conditions and progressing to the desired final time or spatial domain, NDSolve integrates the equations over the specified range. It returns the numerical solution, often as interpolating function objects, which can be used for analysis and visualization. Mathematica's built-in functions can create plots and visual representations of the solution. The technique also provides information about the accuracy of the solution, including error estimates.

Each graph presented in this section offers a comparative analysis of the behavior of hybrid (MoS_2_ + Ag + water) and nanofluid (MoS_2_ + water) solutions. The nanofluid consists of MoS_2_ nanoparticles suspended in a water base fluid, while the hybrid nanofluid incorporates two types of nanoparticles: MoS_2_ and Ag, both dispersed in a water base fluid. Throughout the comparative study, *ϕ*_np1_ = 0.05 = *ϕ*_np2_ is kept constant for the MoS_2_ + Ag + water case, whereas *ϕ*_np1_ = 0.1 and *ϕ*_np2_ = 0 are fixed for MoS_2_ + water. The results are visually depicted using solid lines for hybrid nanofluid (MoS_2_ + Ag + water) and dashed lines for nanofluid (MoS_2_ + water) solutions. Moreover, Table 1 is constructed for thermophysical properties of nanoparticles and base fluid while Tables 2 and 3 are for regression coefficients of skin friction and Nusselt number respectively.

**Table 2:** Regression coefficients of skin friction 
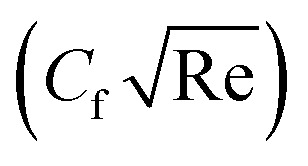
 during multiple quadratic regression analysis for higher estimations of γ*

γ*	*C*_f_	*c*_1_	*c*_2_	*c*_3_	*c*_4_	*c*_5_
**Hybrid nanofluids (MoS**_**2**_ **+ Ag + water)**						
0.1	−17.575	−3.22112	1.97122	−0.294866	−0.075453	0.320673
0.2	−9.51135	−1.72027	1.08551	−0.157455	−0.0457487	0.171275
0.3	−6.76872	−1.20441	0.788643	−0.108862	−0.0359474	0.121005

**Nanofluids (MoS**_**2**_ **+ water)**						
0.1	−17.5709	−3.299	1.9067	−0.304304	−0.0600063	0.326598
0.2	−9.50444	−1.79253	1.02482	−0.169154	−0.0331203	0.174443
0.3	−6.7593	−1.27487	0.728769	−0.12193	−0.0245564	0.122779

**Table 3:** Regression coefficients of Nusselt number 
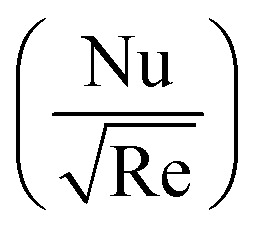
 during multiple quadratic regression analysis for higher estimations of M*

M*	Nu	*d*_1_	*d*_2_	*d*_3_	*d*_4_	*d*_5_
**Hybrid nanofluids (MoS**_**2**_ **+ Ag + water)**						
0.1	−0.0333674	0.603027	0.682105	−0.415122	0.568414	−0.028235
0.2	−0.0332625	0.602356	0.681424	−0.417831	0.562702	−0.0322668
0.3	−0.0331631	0.60172	0.680778	−0.420374	0.557327	−0.036058

**Nanofluids (MoS**_**2**_ **+ water)**						
0.1	−0.0333137	0.602791	0.681885	−0.416022	0.566507	−0.0295857
0.2	−0.0332078	0.602112	0.681195	−0.418731	0.56078	−0.0336241
0.3	−0.0331074	0.601468	0.680541	−0.421278	0.555383	−0.0374266

**Fig. 2:**
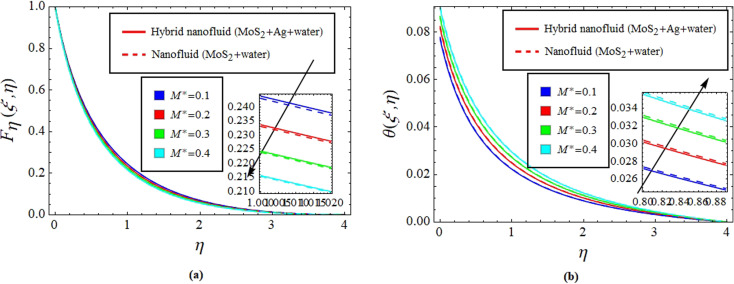
Impact of M* on *F*_*η*_(*ξ*,*η*) (a) and *θ*(*ξ*,*η*) (b).

**Fig. 3:**
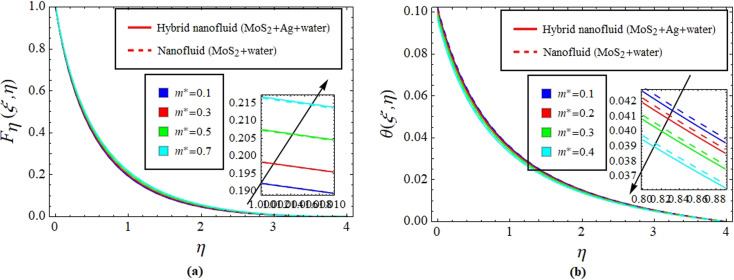
Impact of m* on *F*_*η*_(*ξ*,*η*) (a) and *θ*(*ξ*,*η*) (b).

**Fig. 4:**
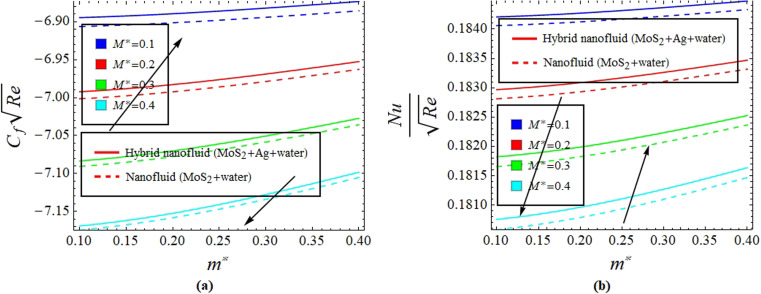
Impact of M* against m* on 
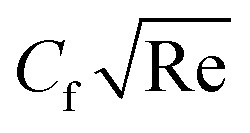
 (a) and 
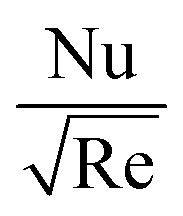
 (b).

**Fig. 5:**
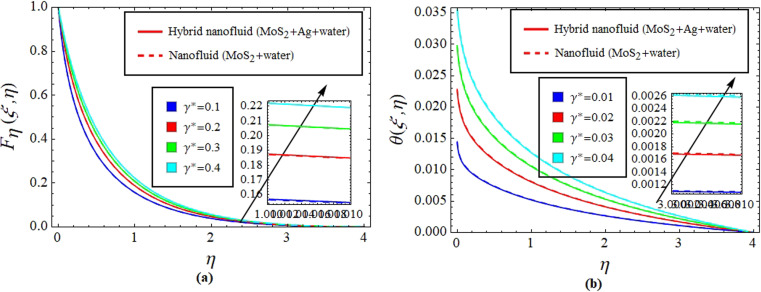
Impact of γ* on *F*_*η*_(*ξ*,*η*) (a) and *θ*(*ξ*,*η*) (b).

**Fig. 6:**
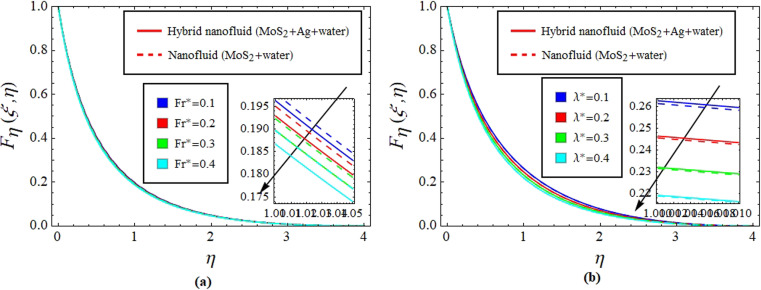
Impact of Fr* (a) and λ* (b) on *F*_*η*_(*ξ*,*η*).

**Fig. 7:**
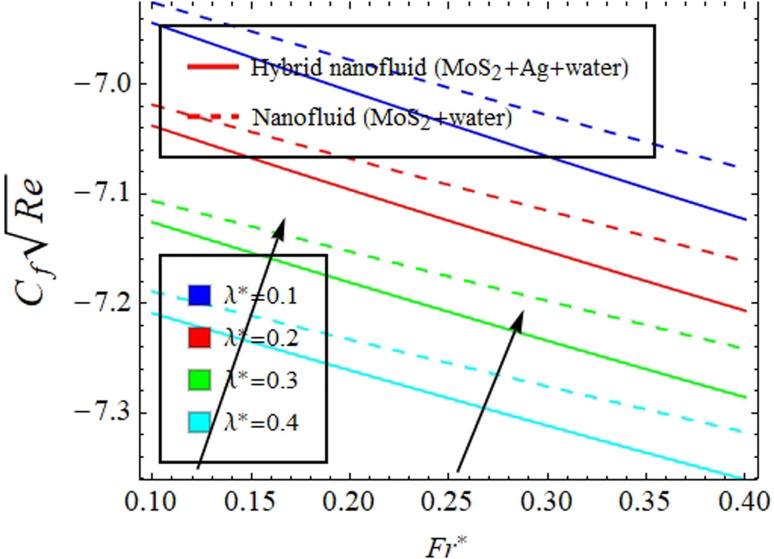
Impact of λ* against Fr* on 
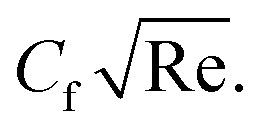

**Fig. 8:**
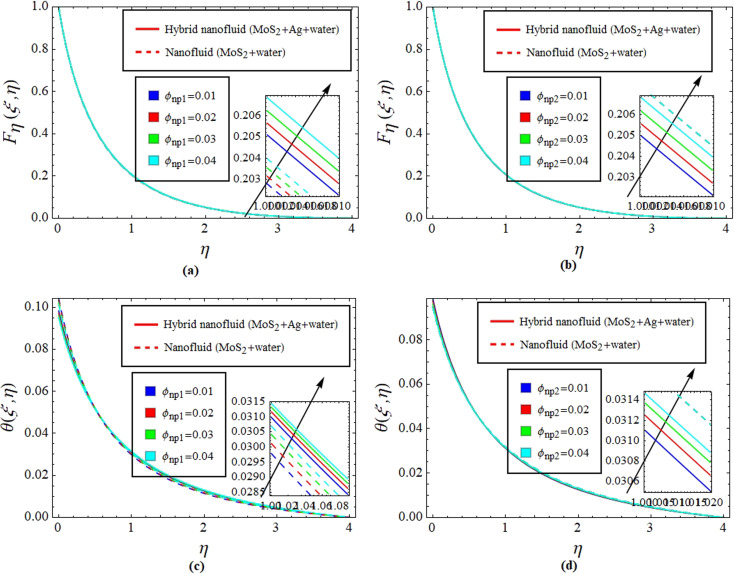
Impact of *ϕ*_np1_ and *ϕ*_np2_ on *F*_*η*_(*ξ*,*η*) (a and b) and *θ*(*ξ*,*η*) (c and d).

**Fig. 9:**
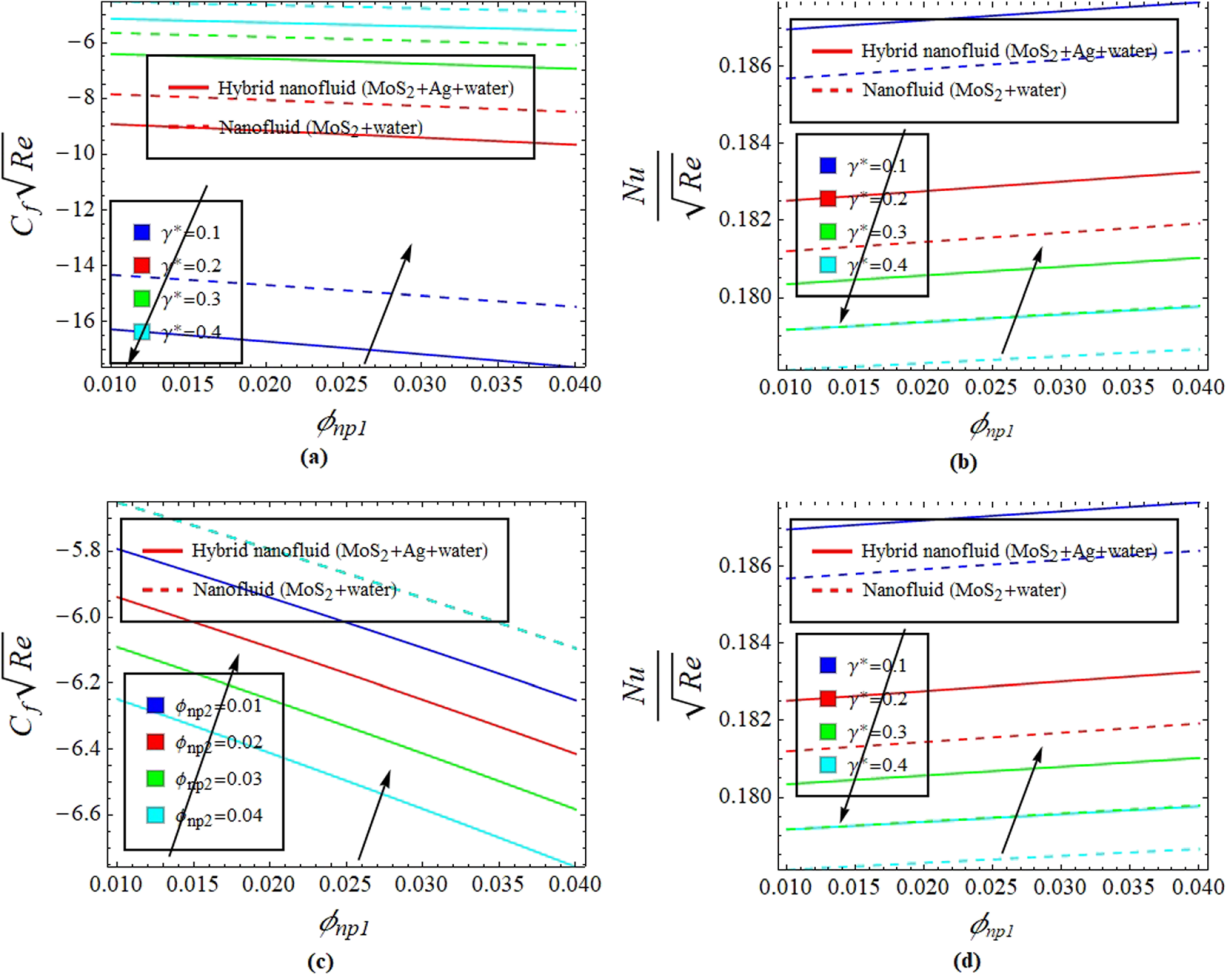
Impact of γ* and *ϕ*_np2_ against *ϕ*_np1_ on 
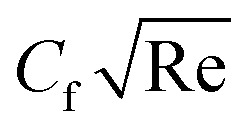
 (a and c) and 
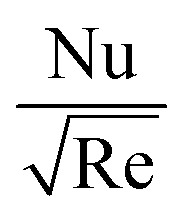
 (b and d).

**Fig. 10:**
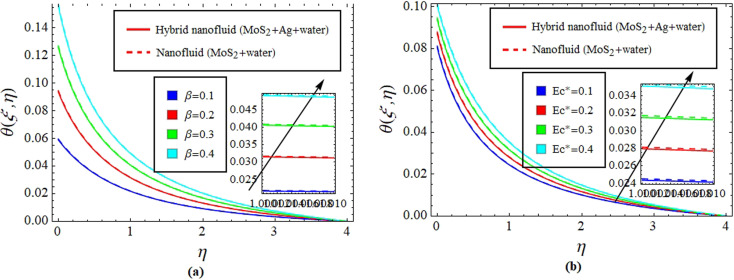
Impact of *β* (a) and Ec* (b) on *θ*(*ξ*,*η*).

**Fig. 11:**
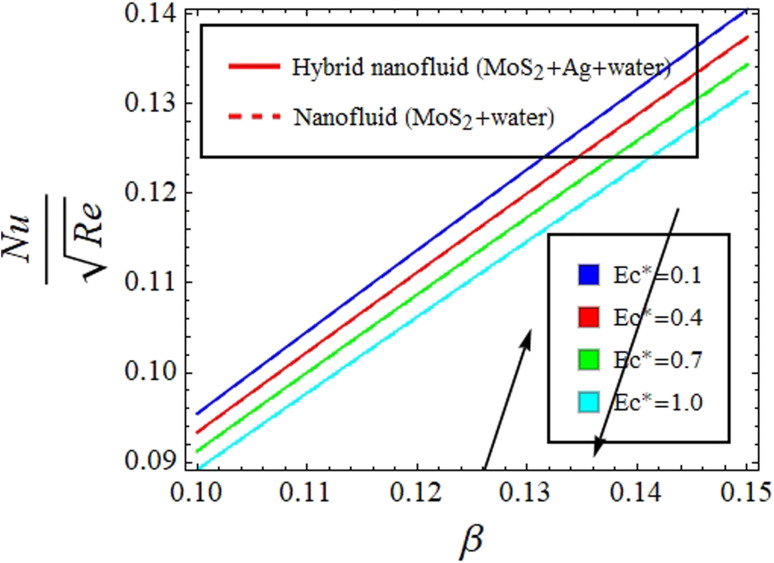
Impact of Ec* against *β* on 
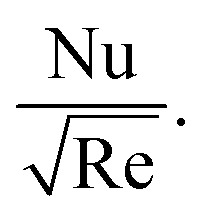

Fig. 2(a) and (b) illustrate the response of M* on *F*_*η*_(*ξ*,*η*) and *θ*(*ξ*,*η*). It is seen that with the variation of M* from 0.1 to 0.4, the opposition force created by *B*_0_ causes the flow field to decline. Physically, this is because particles experience resistance which slows their motion and consequently, lowers *F*_*η*_(*ξ*,*η*), see Fig. 2(a). However, due to *B*_0_, Joule heating promotes the energy transport among the particles. Physically, the system's kinetic energy boosts, which eventually enhances *θ*(*ξ*,*η*), see Fig. 2(b). The behavior is the same for hybrid nanofluid (MoS_2_ + Ag + water) and nanofluid (MoS_2_ + water) solutions. However, the MoS_2_ + Ag + water solution causes a more prominent effect. Fig. 3(a) and (b) depict the outcome of m* on *F*_*η*_(*ξ*,*η*) and *θ*(*ξ*,*η*). The variation is recorded from 0.1 to 0.4. This parameter is generated due to the Hall effect. Physically, it causes charged particles in the fluid to experience a Lorentz force as the particles move through a magnetic field. The fluid's dynamics is affected by this interaction, which leads to an increase in *F*_*η*_(*ξ*,*η*), see Fig. 3(a). Specifically, it amplifies existing driving forces for fluid motion, resulting in accelerated flow in certain regions. On the other hand, the Hall effect's Lorentz force does not only impact *F*_*η*_(*ξ*,*η*) but can also affect *θ*(*ξ*,*η*). When charged particles move through the magnetic field, their trajectories can be altered by the force, leading to changes in *θ*(*ξ*,*η*). This effect can even cause a decrease in *θ*(*ξ*,*η*) in some regions of the fluid due to the redistribution of thermal energy resulting from the interaction between the Lorentz force and the fluid flow, see Fig. 3(b). Fig. 4(a) and (b) depict the impact of M* against m* on 
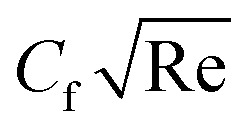
 and 
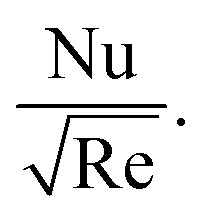
. The variation of M* is from 0.1 to 0.4. The opposing force causes the particles to experience resistance, which is skin drag. Thus, due to variation of M*, 
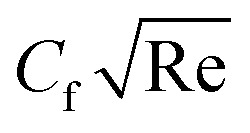
 enhances, but m* causes 
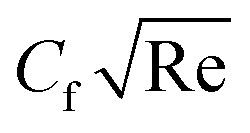
 to lessen, see Fig. 4(a). However, the opposite trend is visible for 
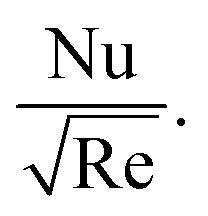
 Physically, due to the complex interplay between the Hall effect and magnetic field the energy transport rate is declined, see Fig. 4(b). Fig. 5(a) and (b) describe the effect of γ* on *F*_*η*_(*ξ*,*η*) and *θ*(*ξ*,*η*). The impression noted suggests that when fluid flows through a curved path, its inertia causes curvature to generate centrifugal forces. These forces are directed away from the center of curvature and can lead to an increase in *F*_*η*_(*ξ*,*η*) on the outer side of the curve, see Fig. 5(a). This increase in velocity often results in higher kinetic energy, which can elevate *θ*(*ξ*,*η*) due to the conversion of kinetic energy into thermal energy through dissipation, see Fig. 5(b). Fig. 6(a) and (b) explain the impact of Fr* and λ* on *F*_*η*_(*ξ*,*η*). Fig. 7 identifies the influence of Fr* against λ* on 
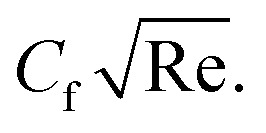
. The Darcy–Forchheimer law expands upon Darcy's law to account for inertial effects in porous media. It highlights that as *F*_*η*_(*ξ*,*η*) decreases, see Fig. 6(a), inertial forces accounted for result in lower flow rates compared to predictions made using Darcy's law (which only considers viscous forces). However, the effect of Fr* on 
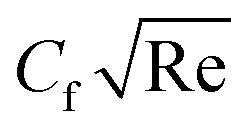
 is more complex. 
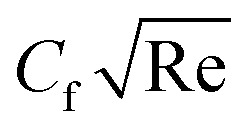
 is determined using the *τ*_w_ at the solid–fluid interface, and both viscous and inertial affect it. Physically, an increase in inertial forces counteract the increase in 
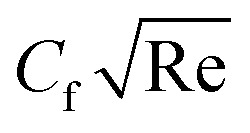
 caused by lower velocity, resulting in a net inclination of 
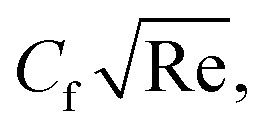
 and see Fig. 7 for the behavior of hybrid nanofluid (MoS_2_ + Ag + water) and for nanofluid (MoS_2_ + water) solutions. However, λ* suggests that due to porosity, there are more spaces available for fluid to flow through. This increased porosity creates additional pathways for fluid movement, resulting in higher *F*_*η*_(*ξ*,*η*). The availability of open spaces allows fluid to move more freely through the medium, leading to greater *F*_*η*_(*ξ*,*η*), whereas λ* results in a lower volume of the solid material within the medium, which reduces the resistance against fluid flow. Therefore, there is less interaction between the fluid and solid surfaces, resulting in 
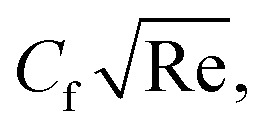
, see Fig. 7. Fig. 8(a)–(d) define the reaction of *ϕ*_np1_ and *ϕ*_np2_ on *F*_*η*_(*ξ*,*η*) and *θ*(*ξ*,*η*) for hybrid nanofluid (MoS_2_ + Ag + water) and for nanofluid (MoS_2_ + water) solutions. The *ϕ*_np1_ notation is for MoS_2_ and *ϕ*_np2_ is for Ag. Due to the increment in *ϕ*_np1_ and *ϕ*_np2_, *F*_*η*_(*ξ*,*η*) is augmented. Physically, by increasing *ϕ*_np1_ and *ϕ*_np2_, *F*_*η*_(*ξ*,*η*) and *θ*(*ξ*,*η*) are heightened due to the enhanced thermal conductivity of the nanoparticles. This increases heat transfer rates within the fluid, creating a temperature gradient that drives fluid motion through thermally induced convection. The energy absorption by the nanoparticles and their transfer to the fluid contribute to increasing *θ*(*ξ*,*η*). Note that the MoS_2_ + Ag + water solution causes a more prominent effect. Thus, an increment in *ϕ*_np1_ and *ϕ*_np2_ improves heat transfer properties and thermally driven fluid motion, leading to enhanced *F*_*η*_(*ξ*,*η*) and *θ*(*ξ*,*η*), see Fig. 8(a)–(d). Fig. 9(a)–(d) provide physical significance of γ* and *ϕ*_np2_ against *ϕ*_np1_ on 
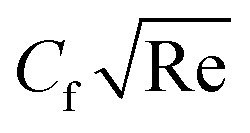
 and 
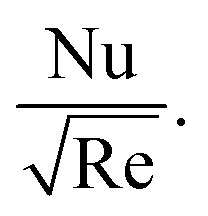
 The graphs are designed for hybrid nanofluid (MoS_2_ + Ag + water) and for nanofluid (MoS_2_ + water) solutions. As γ* enhanced *F*_*η*_(*ξ*,*η*), see Fig. 5(a), it causes a reduction in opposition forces, see Fig. 9(a). Therefore, 
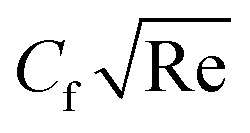
 declines but due to *ϕ*_np1_, 
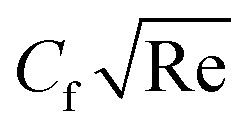
 is boosted up. Similarly, the heat transfer rate is reduced due to an augmentation in γ*, see Fig. 9(b). The effect of *ϕ*_np1_ causes 
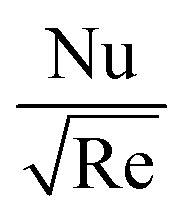
 as well. The physics behind this is that the better thermal conductive properties of MoS_2_ augment 
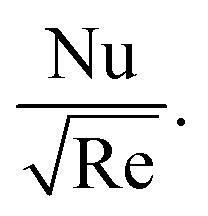
Fig. 10(a) and (b) give explanation of the impact of *β* and Ec* on *θ*(*ξ*,*η*). Fig. 11 identifies the influence of Ec* against *β* on 
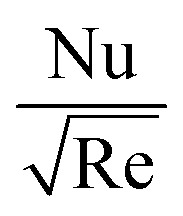
 for hybrid nanofluid (MoS_2_ + Ag + water) and for nanofluid (MoS_2_ + water) solutions. An increment in *β* represents a shift from efficient internal heat transfer to surface resistance as the dominant factor in heat transfer processes 
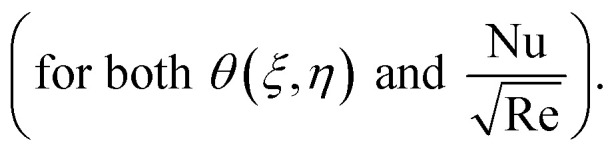
. For *β* ≪ 1, a uniform temperature distribution increment is seen within the material due to rapid internal heat transfer relative to surface resistance. In contrast, *β* ≫ 1 indicates slower internal heat transfer than surface heat transfer, which can result in temperature gradients within the material and notable differences between surface and bulk temperatures, see Fig. 10(a) and 11. Meanwhile, increasing Ec* signifies a greater significance of kinetic energy changes in a fluid than heat transfer rates. This can increase *θ*(*ξ*,*η*), while decreasing 
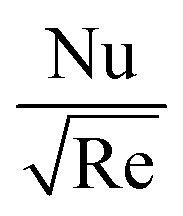
 for hybrid nanofluid (MoS_2_ + Ag + water) and for nanofluid (MoS_2_ + water) solutions, see Fig. 10(b) and 11.

From Table 2, it is seen that the absolute value of the regression coefficient of M* is greater than the regression coefficient of m*. Hence, the effect of M* is prominent over m* on skin friction 
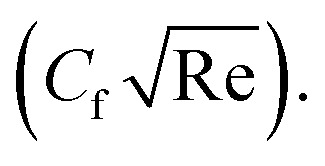
 Similarly, from Table 3, it is observed that the absolute regression coefficient of Ec* is more than that of γ*. Therefore, it is concluded that Ec* is more effective for Nusselt number 
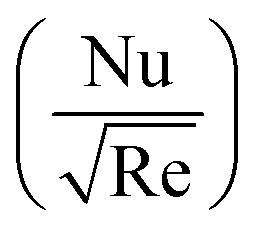
 in comparison with γ*.

## 5. Final remarks

This study investigated the complex interactions of a hybrid nanofluid flowing over a curved surface subjected to stretching. A magnetic field perpendicular to the flow and directed inward engaged with a composite of molybdenum disulfide and argentum nanoparticles suspended in a pure water solution, forming a hybrid nanomaterial. The study analyzed heat transport through magnetohydrodynamics, Darcy–Forchheimer porous medium flow, Joule heating, and a convective boundary condition. Using numerical and statistical methods, the study gained valuable insights into the behavior of the hybrid nanofluid system under specified conditions. The main key points are:

❖ The MoS_2_ + Ag + water solution caused a more prominent effect than MoS_2_ + water.

❖ Joule heating promoted the energy transport among the particles, while Lorentz force caused the flow field to lessen, resulting in an increment in skin friction.

❖ The Hall effect affected fluid's dynamics by increasing velocity, while decreasing skin friction. However, thermal transport declined but the Nusselt number boosted up.

❖ Curvature augmented velocity and causes conversion of kinetic energy into thermal energy, which inclined thermal transport.

❖ The volume fraction improved heat transfer properties and thermally driven fluid motion.

❖ Darcy–Forchheimer flow included viscous and inertial forces which resulted in higher flow rates and diminished skin friction.

❖ Porosity declined flow and enhanced skin friction.

❖ The Biot number caused uniform temperature distribution within the material due to rapid internal heat transfer relative to surface resistance.

❖ The biot number augmented the heat transfer rate significantly.

❖ The Eckert number caused heat capacity to enhance and elevated thermal transport; however, it lessened the Nusselt number.

❖ Regression analysis suggested that the effect of the Eckert number on the Nusselt number was more than that of the Biot number.

❖ The impact of Darcy–Forchheimer flow through regression analysis was prominent over the porosity parameter on skin friction.

### 5.1 Limitations and scope of future work

The study's use of numerical and statistical methods demonstrates its potential for insightful analysis. However, experimental validation is necessary to enhance the model's credibility further and ensure its practical application accuracy. Although the findings are specific to the investigated parameters, they provide a valuable basis for comprehending the behavior of hybrid nanofluids and allow for customized applications in a broad range of scenarios. Real-world complexity, including factors such as turbulence and impurities, emphasizes the importance of future research to expand on this foundation, considering the intricate nature of dynamics of fluid in various environments.

## Conflicts of interest

The authors declare that there is no conflict of interest amongst them.

## Abbreviations

### Nomenclature for the involved expressions

*U*(*r*,*z*) and *W*(*r*,*z*): Velocity components
*F*_*η*_(*ξ*,*η*): Non-dimensional velocity
*U*_0_
: Reference stretching velocity
*θ*(*ξ*,*η*): Non-dimensional temperature
*T*_w_
: Surface/wall temperature
*e*_1_
: Electron charge
*L*
: Characteristic length
Fr*: Forchheimer parameter
*B*_0_
: Magnetic field strength
γ*: Curvature parameter
m*: Hall parameter
Re: Reynolds number
*τ*_w_ = *τ*_*rz*_: Wall shear stress
λ*: Porosity parameter
*r*,*z*: Coordinates
*ψ*(*r*,*z*): Stream function
*U*_w_(*x*): Stretching velocity
*T*(*r*,*z*): Temperature
*T*_∞_
: Ambient temperature
*ne*_1_
: Free electron density
*a*_0_
: Radius of a curved surface
*C*_b_
: Drag coefficient
M*: Dimensionless magnetic parameter
Pr: Prandtl number
Ec*: Eckert number
Nu: Nusselt number
*C*_f_
: Skin friction
*k*_p_
: Porous medium permeability

### Thermophysical features (MoS_2_, Ag, and water)

Ag: Argentum
*μ*_hnf_
: Dynamic viscosity
*ρ*_hnf_
: Density
*κ*_hnf_
: Thermal conductivity
*κ*_np1_
: MoS_2_ thermal conductivity
*ϕ*_np1_
: MoS_2_ volume fraction
(*c*_p_)_hnf_: Specific heat
*σ*_hnf_
: Electrical conductance
*ν*_hnf_
: Kinematic viscosity
*α*_hnf_
: Thermal diffusivity
MoS_2_: Molybdenum dioxide
*μ*_bf_
: Base fluid dynamic viscosity
*ρ*_bf_
: Base fluid density
*κ*_bf_
: Base fluid thermal conductivity
*κ*_np2_
: Ag thermal conductivity
*ϕ*_np2_
: Agvolume fraction
(*c*_p_)_bf_: Base fluid specific heat
*σ*_bf_
: Base fluid electrical conductivity
*ν*_bf_
: Base fluid kinematic viscosity
*α*_bf_
: Base fluid thermal diffusivity

## Note added after first publication

This article replaces the version published on 1st December 2023, which contained errors in equation 14.

## Supplementary Material
